# Knowledge, Perceptions, and Practices of Electronic Waste Management among Consumers in Kampala, Uganda

**DOI:** 10.1155/2021/3846428

**Published:** 2021-02-12

**Authors:** Rebecca Nuwematsiko, Frederick Oporia, Juliet Nabirye, Abdullah Ali Halage, David Musoke, Esther Buregyeya

**Affiliations:** ^1^Department of Disease Control and Environmental Health, School of Public Health, Makerere University College of Health Sciences, Kampala, Uganda; ^2^Department of Health Policy, Planning and Management, Makerere University College of Health Sciences, Kampala, Uganda

## Abstract

**Background:**

Although proper management of electronic waste (e-waste) is key to preventing disease and protecting the environment, there is no clear mechanism for its management in Uganda. This study assessed knowledge, perceptions, and practices of e-waste management among consumers in Kampala city, Uganda.

**Methods:**

We conducted a cross-sectional study among people who used, repaired, or sold electronics (consumers). Both quantitative and qualitative methods of data collection using a sequential explanatory strategy were utilized. The quantitative survey involved 640 study participants, while qualitative interviews included 18 key informant interviews with stakeholders and six focus group discussions with 57 consumers. Modified Poisson regression was used to establish associations with corresponding 95% confidence intervals, and qualitative data analysed thematically.

**Results:**

Two-thirds (67.7%; 433/640) of electronics consumers had poor knowledge on the management of e-waste. More than three-quarters 79.1% (506) of the consumers had positive perceptions towards e-waste management. Consumers perceived e-waste as harmful to human health and the environment. Participants in informal employment were 0.96 times less likely to have positive perceptions towards e-waste management compared to those in formal employment (adjusted PR = 0.96, 95% CI: 0.93–0.99). Mobile phones and televisions were the most owned e-waste with only 7.96% (18/226) and 13.2% (7/53) disposed off, respectively. Selling e-waste to repair shops and donation were the common disposal options.

**Conclusion:**

Knowledge on proper e-waste management is poor among electronic consumers in Kampala, Uganda, though most have positive perceptions. There is need for increased awareness on e-waste management to prevent its effects on health and the environment. Special attention should be towards sensitisation on e-waste handling practices before disposal and final disposal options available.

## 1. Introduction

Electronic waste (e-waste) is growing fast worldwide and is one of the new environmental threats attributed to technological advancements, urbanization, industrialization, increasing population, and economic development [1, 2]. Consequently, these developments come with high use of electronic equipment to sustain them followed by large volumes of waste generated thereafter [2–4]. E-waste is waste generated from any equipment running on electricity or a battery including computers, laptops, televisions (TVs), digital video disc (DVD) players, mobile phones, MPEG-1 audio layer III (MP3) players, and many others which have been disposed by their original users [1]. E-waste also includes a broad range of electronic devices from large household appliances to personal products such as handheld cellular phones, personal stereos, consumer electronics, and computers [5].

An estimated 44.7 million metric tonnes of e-waste were generated globally by 2016 with 2.2 metric tonnes in Africa, out of which only 20% were recycled through appropriate channels [6]. It is estimated that, by the year 2021, the amount of e-waste generated globally will increase to 52.2 million metric tonnes [6]. An assessment carried out in Uganda showed that Government, academic institutions, and Non-Governmental Organisations (NGOs) own the highest number of Information, Communication, and Technology (ICT) equipment in the country at 74.3%, followed by those using them for business purposes at approximately 18.6% and 7.1% for household and personal use [4]. The huge numbers of ICT equipment will eventually become e-waste in the near future and hence there is a need for better planning. Furthermore, in an effort to make ICT affordable, the government of Uganda put tax waivers on importation of computers which promotes the importation of used and refurbished devices such as computers, laptops, and mobile telephones [4].

E-waste is unique because of the toxic, hazardousness, and nonbiodegradable nature of its components [1]. E-waste contains more than 1,000 different substances such as lead, mercury, arsenic, cadmium, selenium, hexavalent chromium, and flame retardants that create dioxins emissions when burned [1, 5]. Poor handling, recycling, and disposal of e-waste can cause severe impacts on public health and the environment [6]. Toxins arising from e-waste have been found to cause brain damage, birth defects, allergic reactions, and cancer [2, 7]. Dismantling, material recovery, and final disposal of e-waste are the source of major environmental and human health impacts [8]. Children, foetuses, pregnant women, elderly people, people with disabilities, workers in the informal e-waste recycling sector, and waste scavengers are at high risk for effects of e-waste. Children and foetuses have an increased risk for effects of e-waste because of their developmental vulnerabilities and compromised systems which are unable to excrete some toxic materials [8–10].

Proper management of e-waste includes reuse, regulated recycling, material recovery, incineration, and landfilling [1, 2]. The choice of a disposal method is however dependant on the available capacity in different countries. Currently, most of the e-waste produced in low and middle-income countries is unregulated and managed by the informal sector through crude means such as informal dismantling, unprofessional recycling, open dumping, unmonitored disposal in landfills, and open burning, predisposing the public to hazardous effects of e-waste [1, 6, 11, 12]. These disposal practices are worse in countries like Uganda where there is no clear management system and regulatory framework for e-waste [13]. In most developed countries, e-waste is shipped to developing countries like India and Ghana to avoid the high expense of proper disposal [2, 8, 11]. The Basel Convention on the control of transboundary movements of hazardous wastes and their disposal was instituted in 1992 to reduce importation of hazardous waste, and many countries including Uganda became members [5]. However, the Basel Convention does not regulate on second hand items and some e-waste scrap which has given room to developed countries to continue shipping e-waste to developing countries which do not even have capacity to dispose it off properly.

Uganda developed the e-waste Management Policy to guide, promote, and ensure the safe management of e-waste in Uganda while contributing to reduction of environmental degradation [14]. A strategy and guidelines for implementation of the policy were also developed to that effect. The guidelines recommend disposal of e-waste in specialised cells or sections in a licensed landfill site while prohibiting burying and incineration because the existing incineration facilities in the country are not suitable for the purpose [15, 16]. These e-waste regulations for Uganda were released in 2011, and nine years later, e-waste disposal and recycling are still not properly done in Kampala city and in other parts of the country. Studies on knowledge, perception, and practices are important in revealing community sensitisation needs consequently influencing evidence based interventions [17]. On the contrary, there is paucity of data on knowledge, perceptions, and practices of e-waste management and the associated negative effects in Uganda. The National Environmental Management Authority (NEMA) has carried out few e-waste awareness initiatives, but these have had limited coverage hence not sufficient to change the knowledge of Ugandans towards proper e-waste management [14]. This study therefore assessed the knowledge, perceptions, and practices of e-waste management among consumers in Kampala so as to provide information to guide appropriate and sustainable interventions.

## 2. Materials and Methods

### 2.1. Study Setting

The study was conducted in three out of the five randomly selected divisions of Kampala City, i.e., Kampala Central, Kawempe, and Makindye. Kampala is the capital city of Uganda and the second most populated urban centre in the country with a total population of 1,507,080 [18]. Kampala City is the business centre for Uganda, and thus it hosts most information and communication technology centres such as the three major telecom companies, and shops that sell majority of the computers brought into the country. According to the Uganda National Household Survey 2016/2017, 88% of households in Kampala own mobile phones individually, 42% own televisions and 13% computers/laptops [18].

### 2.2. Study Design and Population

This was a cross-sectional study carried out between June and September 2019 employing both quantitative and qualitative methods of data collection using a sequential explanatory approach. Qualitative data were collected in addition to get insights into the results and complement findings from the quantitative study. Electronic consumers aged 18 years and above residing and working in Kampala city for at least three months prior to the study were included in the study. We defined electronics consumers as people who used, repaired, or sold electronics. We included both formal and informal workers, because they are deemed to be the biggest consumers of electronics both in terms of use and selling [4]. Specifically, we included workers from the public and private sectors, telecom companies, importers, distributors, private businesses, those involved in repair and recycling of e-wastes, solid waste collection companies, and policy makers. We excluded individuals who did not own any electronic device at the time of data collection.

### 2.3. Sample Size Estimation and Sampling Procedure

We used multistage sampling to randomly select 640 adult electronics consumers who worked and resided in Kampala. The sample size was calculated using the Kish Leslie (1964) formula for cross sectional studies. We considered the following assumptions: a 95% confidence interval (1.96), prevalence, *p* as 50% because no study had been done to estimate the magnitude of the problem, a precision, *α* = 5%, a design effect of 1.5, and a nonresponse rate of 10% [6, 13].

Qualitative data were obtained from purposively selected consumers and representatives from relevant ministries and authorities. We obtained theoretical saturation with six Focus Group Discussions (FGDs) as follows: three with consumers from the informal sector and three with those from the formal sector. Eighteen key informant interviews (KIIs) were also conducted with purposively selected representatives from relevant ministries (the Ministry of Information, Communication, Technology and National Guidance and the Ministry of Health) and authorities (the National Environmental Management Authority and the Kampala Capital City Authority), recyclers, local leaders, and importers of electronics. By the 18^th^ key informant interview, no new information was arising from the participants.

**Figure 1:**
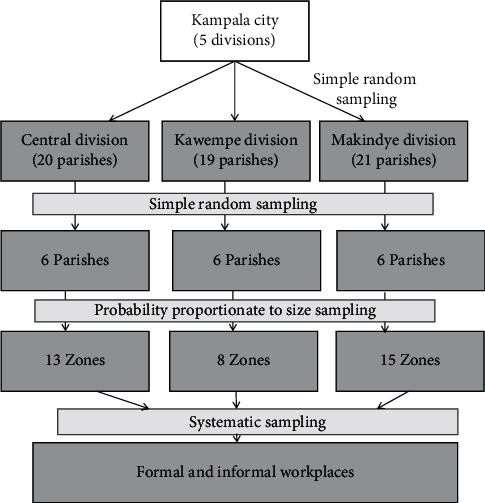
Sampling procedure for the study on knowledge, perceptions, and practices of consumers on e-waste management.

For the quantitative survey, we used a multistage sampling technique from the division in Kampala to the workplace level as shown in Figure 1. Three divisions were randomly selected from the five divisions in Kampala. In each sampled division, simple random sampling was used to select an equal number of parishes to participate in the study. In each selected parish, probability proportional to size was used to determine the zones to include in the study. In each sampled zone, systematic sampling was used to identify workplaces to visit. In each zone, we listed all workplaces with the help of the local chairperson and determined the ‘*k*th' interval, *k* being the total number of workplaces in the zone divided by the sample size of workplaces needed in that zone. We defined a workplace as any room or space where paid work is done. To select a starting point, we stood at the centre of the zone with guidance from the local chairperson. While standing at the centre of the zone, we rolled a pen to determine the direction where to start from. The direction facing the ballpoint of the pen was selected as the starting direction. After listing and numbering the workplaces in the chosen direction, we randomly selected the starting workplace using a table of random numbers. Subsequent workplaces were selected using the “*k*th” number in that direction. In case we reached the other boundary of the zone before completing the sample required, we took another direction after rolling the pen again. At the workplace level, we randomly selected one person to be interviewed. Sampled workplaces with no occupants present at the time of the interview visit were revisited one more time, and if on the second visit no one was present, the next workplace was considered as a replacement. Participants for FGDs and KIIs were purposively selected based on their relevancy to e-waste generation, consumption, policy formulation, control, and management.

### 2.4. Data Collection

The quantitative component utilized a structured questionnaire to obtain data on the knowledge, perceptions, and disposal practices of e-waste and its management. The tool consisted of four parts: (i) sociodemographic profile of the participants: age, sex, marital status, occupation, socioeconomic status, and education. (ii) Knowledge on e-waste and its management: community awareness on e-waste, its effects, availability of legislation, handling, and final disposal options. (iii) Perception on e-waste and its management: how consumers view problems associated with e-waste, recycling, how to handle out of use electronics, and disposal options. (iv) E-waste disposal practices: options of e-waste disposal in the community. We defined electronics as mobile phones, televisions, computers, and refrigerators. The definition was guided by the most owned electronics in the country [4]. The questionnaire was administered to 640 participants by trained research assistants using the Kobocollect toolkit. Participants were interviewed in a suitable location at their places of work.

Focus Group Discussions consisted of electronics consumers in the community. The FGDs were conducted in a suitable place in the community as identified by the participants using an interview guide. The FGDs were modulated by trained research assistants with experience in conducting qualitative interviews. The six FGDs were homogeneously composed consisting of 57 participants with an average of 9 per group lasting at least 1 hour. Of the six FGDs conducted, three had only consumers from the informal sector (1 male, 1 female, and 1 with both male and female participants), and the other three had only consumers from the formal sector (1 male, 1 female and 1 with both male and female participants). We combined both males and female participants in some FGDs so as to gather divergent but complimentary insights on e-waste and its management as viewed by the different gender. During the discussions, an audio recorder was used to capture information and also a note taker was present to take notes. The 18 key informants included representatives purposively selected from Ministry of Information and technology (MICT), Ministry of Health (MoH), National Environmental Management Authority (NEMA), Kampala Capital City Authority (KCCA), Uganda Revenue Authority (URA), Kitezi land fill, recyclers, local leaders, and importers of electronics. The KIIs were conducted in a suitable location at their places of work using interview guides. The KIIs were modulated by trained research assistants with experience in conducting qualitative interviews. During the discussion, an audio recorder was used to capture information, and notes taken. Interview guides for the FGDs and KIIs consisted of follow-on questions on perception on e-waste and its management and disposal options in the community.

### 2.5. Data Management and Analysis

Data collection tools were pretested among six proxy respondents to ensure they yielded the data needed. Meetings were also held at the end of each day to check for consistency, completeness, and also to ensure proper data collection. Data entered in Kobocollect app were exported to Stata 14 (StataCorp, College Station, TX) for cleaning and analysis. We analysed participants' sociodemographics and practices which were then summarised using frequencies and proportions. To determine the level of knowledge, five knowledge questions were scored either 1 for correct or 0 for incorrect responses. Individual knowledge scores were calculated and summed up to give the total score. Participants who had a knowledge score of three and above, ≥3 (range 0–5), were considered to have good knowledge. To determine the level of perceptions of e-waste management, eight perception questions were assessed on a 5-point Likert scale (strongly agree, agree, neutral, disagree, and strongly disagree). Each question was scored depending on the response given with 5 for strongly agree, 4 for agree, 3 for neutral, 2 for agree, and 1 for strongly disagree. The responses were later collapsed to two categories of “agree” for strongly agree and agree responses and “disagree” for neutral, disagree, and strongly disagree. To obtain the overall perception score, all the eight questions were scored and summed and participants whose perception score was ≥24 (range 0–40) were considered to have positive perceptions. Categorisation for the level of knowledge and perceptions was based on Bloom's cut off points.

To measure the association between knowledge, perceptions, and independent variables, we ran a modified Poisson regression via generalized linear models and corresponding 95% confidence intervals while applying a backward elimination method to obtain prevalence ratios (PRs). Prevalence ratios were most preferred over odds ratios because the proportion of our outcome variables was >10%, which would have given biased estimates [19]. Variables that had *p* values of up to 0.2 and those known from literature to be associated with knowledge and perceptions on e-waste management were included in the multivariable model. All inferential statistics were achieved at 95% confidence interval and 5% alpha level.

For qualitative data, all audio tape recordings were transcribed verbatim and translated to English if they were conducted in the local language. Transcripts were then read by two independent members of the study team first to familiarise themselves with the data. This was followed with line by line coding by the two independent people. The independent lists of codes from the two researchers were reviewed to assess intercoder agreement. Any discrepancies were clarified and resolved by comparing each coder's results with raw data until consensus was reached. Coded transcripts were then uploaded into the qualitative analysis software ATLAS.ti Version 7 for thematic analysis using the deductive and inductive approaches. Quotes were then selected to represent the main themes emerging from the study.

### 2.6. Ethical Approval

This study was approved by the Institutional Review Board of Makerere University School of Public Health and registered with the Uganda National Council for Science and Technology. We obtained written informed consent from the participants, and data were treated with maximum confidentiality by storing it in password protected computers only accessed by the research supervisor and principal investigator.

## 3. Results

### 3.1. Sociodemographic Characteristics of Electronic Waste Consumers in Kampala City

A total of 640 participants were interviewed. More than three-quarters 77% (493) of the consumers were aged between 25 and 54 years, with a mean age of 31.99 (SD ± 9.66). Half 50.9% (326) of the participants were females; had secondary level education 263 (41.1%); and were in informal employment 52.7% (337) (Table 1).

### 3.2. Knowledge on Electronic Waste Management among Consumers in Kampala City

Overall, most of the consumers 67.7% (433) had poor knowledge on the management of e-waste. Most of the consumers, 64.8% (415) had good knowledge on the health and environmental effects of e-waste. Poor knowledge was exhibited in the areas of handling of electronics before final disposal 83.3% (533), final disposal options for e-waste 84.8% (543), and e-waste legislation in the country 97.2% (622) (Table 2).

### 3.3. Factors Associated with Knowledge on e-Waste and Its Management

Participants aged ≥55 years were less likely to have good knowledge on e-waste management as compared to those aged ≤24 years from both bivariate (unadjusted PR = 0.82, 95% CI: 0.73–0.91) and multivariable analyses (adjusted PR = 0.82, 95% CI: 0.73–0.91) even after adjusting for potential confounders (Table 3).

### 3.4. Perceptions on e-Waste Management among Consumers in Kampala City

Overall, more than three quarters 79.1% (506) of the electronics consumers had positive perceptions towards e-waste management. Positive perceptions were displayed in the areas of storage of e-waste at home is harmful 62% (397); e-waste can be recycled 87.2% (558); e-waste has effects on health 74.7% (478); and e-waste has effects on the environment 81.3% (520). Negative perceptions on e-waste were most displayed in the area of having personal attachments to the electronic equipment even when out of use 62.5% (400) (Table 4).

Qualitative findings on perceptions of e-waste management both in the FGDs and KIIs were a response to follow-up questions on quantitative information regarding e-waste recycling; its effects on both human health and environment; and personal attachments to electronic equipment even when out of use and perceptions on disposal.

From the interviews, some key informants perceived e-waste recycling as causing more harm than good because of lack of capacity in the community to recycle. It was mentioned that many who were recycling were doing it illegally by dismantling the e-waste to remove parts they deemed necessary and thereafter indiscriminately disposed of what they did not need as stated below:

*E-waste recycling is wrong and it is a problem. Nobody in this country has the capacity to recycle e-waste. There is some electronic waste recycling going on but it's informal. Even when recycled, it will work for a week or two. All this is done informally. If you visit any electronics workshop, you realize that they get spoilt electronics and keep removing different parts until it is finally out of use. That is where the misperceived indiscriminate disposal comes in. You find a shell of T.V at the damp site.* (Key Informant 6)

Consumers also perceived e-waste as harmful to the environment and human health, for example, by contaminating water sources, releasing ozone depleting substances, and exploding causing injuries. Consumers perceived that most effects would be as a result of indiscriminate disposal of e-waste and others acknowledged the risk that comes with handling them during repair as stated below:

*Yes. Do you see that television, it can explode like a bomb? In the end the face could be damaged by the exploding tube. Then also the voltage from televisions is dangerous. We operate and repair them but we are at risk. Secondly, if you got a computer and threw it in the swamp then the water in the swamp will be contaminated and the water will end up somewhere and people consume it. You also know that fridges have ozone depleting substances in them and cause a lot of problems.* (Key Informant 9)

Personal attachments to electronic equipment even when out of use were mentioned by most FGD participants and key informants as a negative perception which prevented consumers from disposing off e-waste. The personal attachments mentioned as preventing disposal of e-waste were for memories especially when the electronics were given by a loved one or because of the significance behind its purchase such as being a first item owned or a special brand as illustrated below:

*Yes. These things (e-waste) are kept depending on the way they got them. My daughter got married and gifted me with a flat screen TV. I have never watched it because I have many TVs. Another person can also keep some thing because their uncle gifted them. So we have that mentality of keeping the e-waste with attachment. These TVs that I buy can be easily disposed of but those gifted electronics are hard to let go*. (Key informant 4)

Regarding disposal of e-waste, there were negative perceptions with some perceiving burning of e-waste as the best option as expressed in the quote below:

*They (e-waste) damage the environment because most of the televisions have plastic casing which when rested on the soil, nothing can be grown there. That way it is spoiling the environment just like polythene bags, it is better they are burnt instead of thrown on the soil.* (informal mixed FGD Participant 7)

### 3.5. Factors Associated with Perceptions of e-Waste and Its Management

From the bivariate analysis, those in informal employment were less likely to have positive perceptions towards e-waste and its management compared to those in formal employment (unadjusted PR = 0.96, 95% CI: 0.92–0.99). After adjusting for confounders, the same factor remained statistically significant. E-waste consumers in informal employment were 0.96 times less likely to have positive perceptions towards e-waste and its management compared to those in formal employment (adjusted PR = 0.96, 95% CI: 0.93–0.99) (Table 5).

### 3.6. Disposal Practices of e-Waste among Electronics Consumers in Kampala

**Figure 2:**
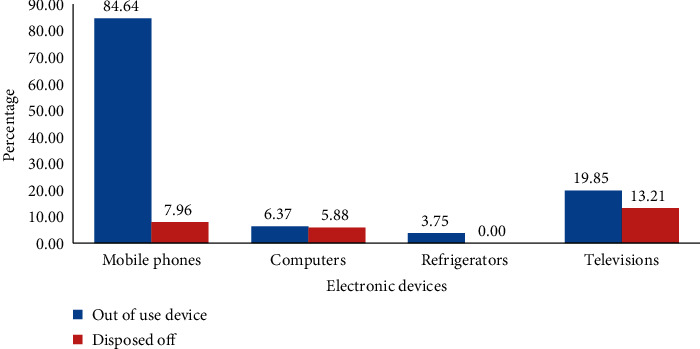
Disposal of e-waste among consumers in Kampala.

Mobile phones (84.6% (226/267)) and televisions (19.9%) were the most owned e-waste out of which only 7.96% (18/226) and 13.2% (7/53) were disposed off, respectively. None of the refrigerators were disposed off (Figure 2). Out of the 26 consumers who reported disposing off their e-waste, 10 sold them off to repair shops, an equal number (7) donated and dumped them in the general waste, and the other two burnt them.

Regarding the undisposed e-waste, most of them 93.2% (261/280) were kept in the consumer's homes followed by storage at the mechanic 2.1% (6/280) and a few were at the workplace (0.7% (2/280)).

Follow-up qualitative findings on why e-waste was stored in the consumer's homes revealed that most consumers kept the undisposed e-waste at home majorly because they had personal attachments to them. Most personal attachments were around love for the electronics as mentioned below:

*Having personal attachments is usual. Even when growing up, our grandparents had their things that they did not want anyone to touch. Even when it got spoilt, they would not let you touch it. You would get beaten for touching ‘grandpas' old spoilt radio because he liked it so much……. That is the character we grew up seeing. It has sentiments attached.* (informal female FGD Participant 11)

A big number of FGD participants also mentioned that they stored e-waste in their homes to act as toys for children to play with as illustrated below:

*We usually have a station point where we dispose the e-waste of but sometimes when the* waste *collectors do not turn up, we can give it to a child to play with. But to say the truth, even me as an individual, I do not dispose them of the right way….* (informal mixed FGD Participant 8)

Having no knowledge of how to dispose e-waste and no designated place for disposal was also expressed by some consumers as the reason as to why they have kept the e-waste in their homes as described in the quote below. Key informants from regulatory bodies however mentioned that plans were underway to put up an e-waste collection and disposal site in a district near Kampala which is far away from human settlement.

*Yes the consumers currently store them (e-waste) because they do not know what to do. They do not have any knowledge on how to dispose them. For example I have a radio that my dad first bought before I was born. I cannot throw it so I just keep it and look at it and the best I can do is to tell my children about it.* (Key Informant 16)

## 4. Discussion

This study assessed consumers' knowledge, perceptions, and practices on e-waste and its management in Kampala, Uganda. In the study, electronics consumers had poor knowledge on the management of e-waste. More than three-quarters of the consumers had positive perceptions towards e-waste management. Consumers perceived e-waste as harmful to the environment and human health. Participants in informal employment were 0.96 times less likely to have positive perceptions towards e-waste management compared to those in formal employment. Few e-waste were disposed, and the rest were kept in homes and offices due to personal attachments, limited knowledge on disposal and lack of a disposal site. Selling electronics to repair shops and donation were the common disposal options.

Our findings demonstrate the need to have in place measures to improve e-waste management in Kampala to be able to protect human health and the environment.

Most e-waste consumers had poor knowledge on e-waste management specifically on the disposal and handling practices. This is similar to findings from India where most people were unaware of e-waste management [20–24]. Most consumers having poor knowledge on e-waste management is attributed to the fact that there are few sensitisation sessions on e-waste in Kampala and even those carried out are only to the public sector employees. More so, lack of a formal e-waste management system further denies the consumers a chance to know proper e-waste management. Due to lack of knowledge on e-waste management, people blindly expose their lives to the damaging effects of e-waste, some for economic reasons leading negative health effects which would have been prevented [9, 10, 25]. However, most participants in our study had good knowledge on the effects of e-waste. This finding is similar to other studies where most consumers were aware of the effects of exposure to e-waste [21, 22, 26]. Knowledge on the effects of e-waste leads to proper handling and disposal. Furthermore, most consumers did not know the existence of e-waste legislations in the country. This is similar to studies elsewhere where most people were unaware of the existing e-waste legislations [20]. Lack of knowledge on the legislations may be because they are not implemented and also there is limited awareness of their existence by the responsible authorities. This contributes to the low level of knowledge on e-waste management among consumers. Awareness of e-waste legislations could promote proper e-waste management behaviours consequently preventing the effects that could arise from poor management. There is therefore a need for the responsible government authorities and civil society organisations to raise awareness of e-waste management and the legislations in place so as to enable proper handling and disposal among consumers.

More than three-quarters of the consumers had positive perceptions towards e-waste management. This agrees with findings from elsewhere where most consumers had positive perceptions towards e-waste management [2, 27–29]. High positive perceptions registered among the consumers could be due to the increased health consciousness in the population promoted through sensitisation platforms for other health risk factors like use of plastics, leaded paint, and inappropriate use of mobile phones [20, 30]. Positive perceptions among consumers is an opportunity to foster proper management practices since the population already has some good intention and belief to appropriately manage the e-waste. E-waste consumers in informal employment were 0.96 times less likely to have positive perceptions towards e-waste and its management compared to those in formal employment. This could be because most workers in the informal sector have limited education and hence may be compromised in their knowledge and exposure to information on e-waste which consequently affects their perceptions [23, 27, 31, 32]. Having targeted interventions to consumers in informal employment may yield positive results to improve proper e-waste management. The informal sector is often left out in interventional research due to the complexity of their working conditions; yet, they constitute the largest number of workers and are also more likely to engage in risky health behaviours since there is limited monitoring [4]. Sensitisation campaigns should therefore target those in informal employment, given that they displayed negative perceptions towards e-waste management.

Few e-wastes were disposed off, and the common disposal options were selling to repair shops and donation to people who needed the electronics. This finding is similar to studies elsewhere in India, Ghana, and Nigeria where most e-waste was sold off to scrap dealers and recyclers, donated, or kept at home [22, 29, 33, 34]. Selling off and donation of e-waste provide the most feasible disposal option in the absence of a formal management system. Selling and donation of e-waste prevents them from accumulating unnecessarily consequently causing health effects and harbouring rodents. Most of the undisposed e-waste was kept in consumer's homes for various reasons such as acting as toys for children, because of personal attachments, limited knowledge on disposal, and lack of a disposal site. Keeping e-waste in homes was also documented by studies elsewhere in India, Ghana, and Nigeria [22, 29, 33, 34]. Exposing children to play with e-waste could be detrimental to their health as they are more vulnerable to the effects from e-waste components given that their systems are still developing and may not easily excrete the toxins they are exposed to [4, 7, 8]. It is therefore paramount that the Government of Uganda speeds up the process of establishing an e-waste disposal site in Kampala so that consumers can dispose off the e-waste that may be available in the homes and offices of the population. Establishment of a disposal site should however be backed up with sensitisation on the proper handling and disposal of e-waste with emphasis on its negative effects so as to reduce indiscriminate disposal and consumer's personal attachments to the e-waste.

To the best of our knowledge, this is the first study on knowledge, perceptions, and practices of e-waste management among consumers in Uganda. Most studies have focused on general solid waste management ignoring e-waste; yet, it is hazardous and could jeopardise all the efforts done towards disease prevention through proper solid waste management. Triangulation of information from different data collection methods was a strength to our study since it gave us more insight into the quantitative findings but also increased the validity of the results. Our study therefore provides important findings that can be used to guide the design of an implementation strategy for raising community awareness on e-waste management. As plans are underway to establish an e-waste collection and disposal facility in Kampala, findings from this study may also inform the collection strategy and who to target as champions for that cause. However, our study was based on self-reported data from the respondents which could have introduced a social desirability bias since human beings always want to be seen doing the right thing as opposed to what exactly is practiced. This was counteracted by triangulation of quantitative and qualitative data which increased the validity and reliability of our findings.

## 5. Conclusion and Recommendations

Electronics consumers in Kampala had poor knowledge on handling of e-waste, existing legislations and final disposal with some recommending burning and others disposal in the general waste. Most consumers had positive perceptions towards e-waste management. Consumers perceived e-waste as harmful to human health and the environment. With most electronics consumers exhibiting limited knowledge on e-waste management, all relevant stakeholders led by Ministry of Information and technology (MICT) should intensify awareness campaigns to address the current gap. Sensitisation messages should emphasize available e-waste handling practices such as reuse, repair, recycle, and good practices for final disposal. The existing legislations in place should also be made known and available to the public to guide their e-waste management practices. Increase in knowledge on e-waste management will consequently improve perceptions. Few out of use electronics were disposed, and the rest kept in homes and offices due to emotional attachments, poor knowledge on disposal and lack of a disposal site. The process of establishing a formal e-waste management system in Uganda should therefore be expedited so as to give guidance on how to appropriately handle the waste and also provide collection and disposal facilities for the consumers. Establishment of a disposal site will relieve consumers of the piles of e-wastes stored. Disposal of the e-waste stored in homes and offices will consequently reduce the risks posed by exposure to these wastes and prevent the negative effects that could arise.

## Figures and Tables

**Table 1:** Sociodemographic characteristics of electronic waste consumers in Kampala city.

Variable	Frequency (*n* = 640)	Proportion (%)
*Division of stay*		
Makindye	241	37.66
Kawempe	200	31.25
Central	199	31.09
*Age (years)*		
≤24	127	19.84
25–54	493	77.03
≥55	20	3.13
Mean = 31.99 (9.66)		
*Sex*		
Male	314	49.06
Female	326	50.94
*Marital status*		
Single	254	39.69
Married	364	56.87
Divorced/widowed	22	3.44
*Education status*		
No formal education	20	3.13
Primary	125	19.53
Secondary	263	41.09
Tertiary	232	36.25
*Occupation*		
Formal employment	303	47.34
Informal employment	337	52.66

**Table 2:** Level of knowledge on electronic waste management among electronic waste consumers in Kampala city.

E-waste knowledge items	Good	Poor
Overall knowledge score	207 (32.34)	433 (67.66)
	Mean = 2.28, SD = 0.83	

	Yes	No
Knowledge on at least one example of e-waste	639 (99.84)	1 (0.16)
Knowledge on health and environmental effects of e-waste	415 (64.84)	225 (35.16)
Knowledge on handling electronics before final disposal (reuse, repair, recycle, donate)	107 (16.72)	533 (83.28)
Knowledge on final disposal options for e-waste	97 (15.16)	543 (84.84)
Knowledge on e-waste legislation in the country	18 (2.81)	622 (97.19)

Note: knowledge was assessed by giving 1 to a correct response and 0 to wrong response. The scale classified knowledge as Good with score ≥3 (range 0–5) and Poor <3.

**Table 3:** Factors associated with knowledge of e-waste management among consumers in Kampala city.

Variable	Good knowledge *n* (%)	Poor knowledge *n* (%)	Unadjusted PRRs	Adjusted PRRs	*p* value
*Division of stay*					
Central	68 (34.2)	131 (65.8)	1.0	1.0	
Kawempe	58 (29.0)	142 (71.0)	0.96 (0.89, 1.03)	1.00 (0.93, 1.08)	0.977
Makindye	81 (33.6)	160 (66.4)	0.99 (0.93, 1.06)	0.99 (0.93, 1.07)	0.979

*Age (years)*					
≤24	36 (28.3)	91 (71.7)	1.0	1.0	
25–54	170 (34.5)	323 (65.5)	1.05 (0.98, 1.21)	1.04 (0.97, 1.13)	0.861
≥55	1 (5.0)	19 (95)	0.82 (0.73, 0.91)*∗*	0.82 (0.73, 0.91)*∗∗*	**<0.001**

*Sex*					
Female	99 (30.4)	227 (69.6)	1.0	1.0	
Male	108 (34.4)	206 (65.6)	1.03 (0.98, 1.09)	1.03 (0.98, 1.09)	0.296

*Marital status*					
Divorced/widowed	7 (31.8)	15 (68.2)	1.0		
Single	84 (33.1)	170 (66.9)	1.01 (0.87, 1.18)		
Married	116 (31.9)	248 (68.1)	1.00 (0.86, 1.16)		

*Education status*					
None	10 (50.0)	10 (50.0)	1.0	1.0	
Primary	43 (34.4)	82 (65.6)	0.89 (0.76, 1.05)	0.91 (0.77, 1.06)	0.231
Secondary	78 (29.7)	185 (70.3)	0.86 (0.74, 1.01)	0.87 (0.75, 1.02)	0.081
Tertiary	76 (32.8)	156 (67.2)	0.89 (0.76, 1.03)	0.89 (0.77, 1.05)	0.175

*Occupation*					
Formal employment	95 (31.4)	208 (68.6)	1.0		
Informal employment	112 (33.2)	225 (66.8)	1.01 (0.96, 1.07)		

*Level of perception*					
Negative	47 (35.1)	87 (64.9)	1.0	1.0	
Positive	160 (31.6)	346 (68.4)	0.97 (0.91, 1.04)	0.97 (0.91, 1.04)	0.454

*∗p* < 0.05.

**Table 4:** Level of perceptions regarding e-waste management among consumers in Kampala city.

Perception items	Positive	Negative
Overall perception score	506 (79.06)	134 (20.94)
	Mean = 28.25, SD = 5.65	

	Agree	Disagree
Storing e-waste at home is harmful	397 (62.03)	243 (37.97)
E-waste can be recycled	558 (87.19)	82 (12.81)
E-waste recycling is harmful to the environment	319 (49.84)	321 (50.16)
E-waste recycling has effects on human health	336 (52.50)	304 (47.50)
There are health effects associated with e-waste	478 (74.69)	162 (25.31)
E-waste has negative effects on the environment	520 (81.25)	120 (18.75)
Have personal attachments to electronic equipment even when out of use	400 (62.5)	240 (37.5)
E-waste should be disposed with general waste	162 (25.31)	478 (74.69)

Note: the scale classified perception as positive with score ≥24 (range 0–40) and negative <24. The responses were collapsed to two categories of “agree” for strongly agree and agree responses and “disagree” for neutral, disagree, and strongly disagree.

**Table 5:** Factors associated with perceptions of e-waste management among consumers in Kampala city.

Variable	Positive perception *n* (%)	Negative perception *n* (%)	Unadjusted PRs	Adjusted PRs	*p* value
*Division of stay*					
Central	158 (79.4)	41 (20.6)	1.0		
Kawempe	155 (77.5)	45 (22.5)	0.99 (0.95, 1.04)		
Makindye	193 (80. 1)	48 (19.9)	1.00 (0.96, 1.05)		

*Age (years)*					
≤24	100 (78.7)	27 (21.3)	1.0	1.0	
25–54	391 (79.3)	102 (20.7)	1.00 (0.96, 1.05)	1.00 (0.96, 1.05)	0.852
≥55	15 (75)	5 (25.0)	0.98 (0.87, 1.09)	0.98 (0.88, 1.10)	0.763

*Sex*					
Female	265 (81.3)	61 (18.7)	1.0	1.0	
Male	241 (76.8))	73 (23.2)	0.97 (0.94, 1.01)	0.98 (0.94, 1.01)	0.225

*Marital status*					
Divorced/widowed	17 (77.3)	5 (22.7)	1.0		
Single	202 (79.5)	52 (20.5)	1.01 (0.91, 1.12)		
Married	287 (78.8)	77 (21.2)	1.01 (0.91, 1.12)		

*Education status*					
None	15 (75)	5 (25)	1.0		
Primary	93 (74.4)	32 (25.6)	0.99 (0.89, 1.12)		
Secondary	203 (77.2)	60 (22.8)	1.01 (0.90, 1.13)		
Tertiary	195 (84.1)	37 (15.9)	1.05 (0.94, 1.18)		

*Occupation*					
Formal employment	252 (83.2)	51 (16.8)	1.0	1.0	
Informal employment	254 (75.4)	83 (24.6)	0.96 (0.92, 0.99)^*∗*^	0.96 (0.93, 0.99)^*∗∗*^	**0.021**

*Level of knowledge*					
Poor	346 (79.9)	87 (20.1)	1.0	1.0	
Good	160 (77.3)	47 (22.7)	0.99 (0.95–1.02)	0.99 (0.95, 1.02)	0.481

## Data Availability

The data used to support findings of this study may be released upon request to the Institutional Review Board of Makerere University School of Public Health. They can be contacted at hdrecadmin@musph.ac.ug.
